# Enhancing Lyme Disease Surveillance by Using Administrative Claims Data, Tennessee, USA

**DOI:** 10.3201/eid2109.150344

**Published:** 2015-09

**Authors:** Joshua L. Clayton, Stephen G. Jones, John R. Dunn, William Schaffner, Timothy F. Jones

**Affiliations:** Centers for Disease Control and Prevention, Atlanta, Georgia, USA (J.L. Clayton);; Tennessee Department of Health, Nashville, Tennessee, USA (J.L. Clayton, J.R. Dunn, T.F. Jones);; Blue Cross Blue Shield of Tennessee, Chattanooga, Tennessee, USA (S.G. Jones);; Vanderbilt University School of Medicine, Nashville (W. Schaffner)

**Keywords:** Lyme disease, Borrelia burgdorferi, public health surveillance, bacteria, Tennesee, United States

## Abstract

Lyme disease is underreported in the United States. We used insurance administrative claims data to determine the value of such data in enhancing case ascertainment in Tennessee during January 2011–June 2013. Although we identified ≈20% more cases of Lyme disease (5/year), the method was resource intensive and not sustainable in this low-incidence state.

Lyme disease is the most common tickborne disease in the United States, with >36,000 cases reported to the Centers for Disease Control and Prevention (CDC) during 2013 (*1*). Tennessee, a low-incidence state, reported only 25 Lyme disease cases during 2013 (*2*). In addition, *Borrelia burgdorferi*–infected ticks have been identified in only 1 Tennessee county (G.J. Hickling, unpub. data). 

CDC estimates that Lyme disease may be underreported by a factor of 10 (*3*). A study using administrative claims data from a Tennessee health insurance provider similarly estimated that Lyme disease incidence is 7-fold higher than is reported to the Tennessee Department of Health (TDH) (*4*). To determine the usefulness of claims data, which can vary in accuracy (*5**,**6*), we evaluated medical records of persons given a Lyme disease diagnosis in claims data or surveillance in Tennessee.

## The Study

We examined Lyme disease cases reported to TDH and compared them with diagnoses identified from Blue Cross Blue Shield of Tennessee (BCBST) claims data during January 2011–June 2013. BCBST is a health insurance provider covering ≈50% of Tennessee’s population. TDH cases met the national surveillance case definition for Lyme disease (*2*), consisting of the following criteria: clinical (erythema migrans [EM] rash or late manifestation of disease), laboratory (positive results by immunoassay followed by positive western blot results), and exposure and endemicity (possible exposure to infected ticks <30 days before rash onset). A person with physician-diagnosed disease who met laboratory criteria was considered to have a probable case. A person with a confirmed case had an EM rash and either met laboratory criteria, had possible exposure to ticks, or had a late manifestation of disease and positive laboratory results. We defined Lyme disease diagnosis for a BCBST-insured person as assignment of >3 primary or secondary codes for Lyme disease (088.81, International Classification of Diseases, Ninth Revision [ICD-9]), recorded in the claims data.

We used deterministic matching to identify persons in BCBST and TDH data. Medical records of one third of BCBST-insured persons whose cases were not reported to TDH were selected for review. Records were requested from the office visit on the date of Lyme disease diagnosis and for 1 office visit before and after diagnosis. BCBST-insured persons with a Lyme disease diagnosis were then classified according to the case definition (*2*). BCBST-insured persons not meeting the case definition were assigned into the following categories: 1) subsequently ruled out through negative laboratory testing, 2) self-reported or physician-recorded history of Lyme disease (before the study period), or 3) insufficient data for case determination. This analysis was exempted from institutional review board review.

During the study period, ≈3 million Tennessee residents were insured by BCBST, and 391 (0.01%) met criteria for diagnosed Lyme disease. During the same period, TDH received 74 reports of Lyme disease (9 confirmed, 65 probable). Of these, 24 (32%) persons were BCBST-insured at time of diagnosis (Figure). No differences by age and sex were noted between the 391 BCBST-insured persons and 74 TDH case-patients, and most were identified in highly populated counties (Davidson, Hamilton, Knox, Shelby).

**Figure:**
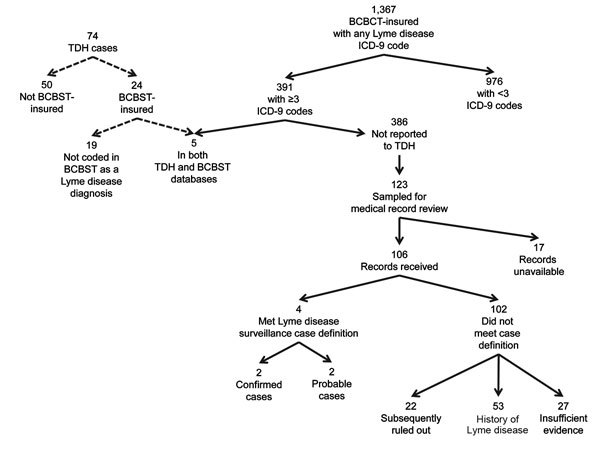
Identification of Lyme disease cases from the Tennessee Department of Health case-based surveillance and Blue Cross Blue Shield of Tennessee administrative claims data, Tennessee, USA, 2011–2013. BCBST, Blue Cross Blue Shield of Tennessee; ICD-9, International Classification of Diseases, Ninth Revision; TDH, Tennessee Department of Health.

Five Lyme disease cases were identified in both BCBST and TDH data, 386 appeared in BCBST data only, and 19 appeared in TDH data only. All 5 matched persons were classified by TDH as having probable cases. Of the 386 persons only in BCBST, 123 were randomly sampled; 106 medical records were reviewed; only 4 (3.8%) met the case definition (2 confirmed, 2 probable). Extrapolating the proportion of true cases (3.8%) identified from this sample, we believe that ≈14 additional cases would have been identified through review of BCBST claims data during the 2.5-year study period. Adding 14 additional cases to the 24 confirmed and probable cases already reported to TDH among BCBST-insured persons, 38 cases would have been identified. Only 19 of the 38 cases would be identified through review of BCBST data (sensitivity 50%). Of 391 BCBST-insured persons with >3 ICD-9 codes for Lyme disease, 19 met the national case definition (positive predictive value 5%).

Of 102 BCBST-insured persons selected for review whose conditions did not meet the case definition, 22 were subsequently ruled out by laboratory testing after the visit in which the diagnosis was coded. For 27, evidence was insufficient to determine case classification, and 53 had a history of Lyme disease (23/53 [43%] had been prescribed antibiotic medications to treat Lyme disease).

Nineteen BCBST-insured persons met the case definition and were reported to TDH as having Lyme disease but were not identified as such in BCBST claims data during the study period. In all instances, no ICD-9 code for Lyme disease was coded in billing records, despite the diagnosis in the medical record and subsequent reporting to TDH. The 4 most frequent ICD-9 codes used for these persons were fever (21%), myalgia/myositis (21%), malaise and fatigue (16%), and gynecologic examination (16%).

## Conclusions

By supplementing passive surveillance with BCBST claims data, we identified 20% more Lyme disease cases than were reported to TDH. The additional cases were diagnosed by clinicians and coded as Lyme disease in administrative claims. In this low-incidence state, most BCBST-insured persons with diagnosed Lyme disease did not meet the case definition, and the positive predictive value of BCBST data was low. The resources required to determine true cases from those diagnosed in BCBST claims data were substantial. Without an improved algorithm for identifying true cases, using these administrative data to supplement health department surveillance would be unsustainable.

Medical records of one fourth of the sample lacked sufficient information for case determination, and records of half showed a history of Lyme disease. Strikingly, none of the persons with a history of Lyme disease had any previous ICD-9 code for Lyme disease recorded by BCBST. Also surprisingly, 8 persons who first appeared to be incident case-patients, according to the BCBST algorithm, had been reported to TDH in the past (for 1 case-patient, >10 years earlier). These previously reported cases decreased the positive predictive value of BCBST data.

Among BCBST-insured persons not meeting the case definition, diagnoses were made by a limited number of clinicians. Understanding how these few clinicians came to diagnose many persons with Lyme disease may aid physician training. Of BCBST-insured persons with a history of Lyme disease, approximately half had current prescriptions for antimicrobial drugs. Although we were unable to assess whether any of these prescriptions represented long-term treatment for a chronic Lyme disease diagnosis, providers and patients should be educated regarding the lack of effectiveness and risks associated with long-term antimicrobial therapy (*7*).

Half of the BCBST-insured persons had a self-reported or physician-recorded history of Lyme disease that could not be verified by our cross-sectional analysis. One quarter of medical records had insufficient information to make a case determination, stemming from a lack of timely and adequate laboratory testing. Whether these data quality deficiencies biased our results is unknown. A history of Lyme disease does not exclude the potential for reinfection (*8*), but the large proportion of persons in this category would be unlikely, given the low incidence of Lyme disease. Southern tick–associated rash illness, caused by *B. lonestari,* produces an EM-like rash and may have confounded our use of administrative claims to identify Lyme disease (*9*).

This study was a special collaboration between TDH and BCBST medical informatics staff and required substantial resources of personnel and time, a level of surveillance not sustainable long-term. Although claims data offer an opportunity for identifying additional Lyme disease cases for public health surveillance, a more efficient means for differentiating cases from noncases is needed before such a system will be practical.
